# What We Know and Don't Know About Lyme Disease

**DOI:** 10.3389/fpubh.2021.819541

**Published:** 2022-01-21

**Authors:** Sam T. Donta

**Affiliations:** Consultant, Infectious Diseases, Falmouth Hospital, Falmouth, MA, United States

**Keywords:** Lyme disease, Lyme diagnosis, Lyme pathogenesis, Lyme treatment, Lyme serology

## Abstract

We know the cause of Lyme disease. We know that the bacteria can be found in the initial rash, and occasionally in the blood in the subsequent 2–3 months, but after then, its subsequent location is unknown. Whereas diagnosis and treatment of early Lyme disease is generally straightforward, the etiology of relapsing or persisting symptoms is yet to be defined, and presents clinical challenges. There are no current tests to determine if the infection is still present or absent, thus complicating diagnosis and treatment. Presented here are approaches to the diagnosis and treatment of persisting Lyme disease, based on available published information, and the experience of the author.

## Introduction

It has been more than 40 years since the discovery of the causative agent of Lyme disease. Much has been learned, but several key questions remain: 1-how do we know if the infection has been eradicated, 2-can it become dormant, then reactivate, 3- in patients with persistent symptoms, are these due to continuing infection or to non-infectious sequelae, and 4-are there treatments that can resolve the infection?

## Pathogenesis

We know that Lyme disease is caused by the bacterium *Borrelia burgdorferi*, transmitted by the bite of an Ixodes tick. We know that the bacteria may be isolated from the typical erythema migrans rash, and can be occasionally recovered from the circulating blood in the subsequent 2–3 months (1). After that time, it has not been possible to consistently isolate the bacteria from any body fluids or tissues.

So, where are they? Under the skin, as demonstrated in studies with macaque primates (2), and similarly in preliminary studies in humans (3)? Intracellularly, as is the case of most, if not all pathogens that can become latent, then recur? Or both? Hence, the central question at the heart of the controversy surrounding the diagnosis and treatment of Lyme disease; i.e., whether persisting or relapsing symptoms are due to continuing infection or due to post-infectious phenomena.

Accumulating evidence regarding the persistence of biologically active, albeit non-replicating bacteria derives from several studies in various animal models. Hodzic et al. demonstrated that *B. burgdorferi* can persist in mice following antibiotic treatment but were non-cultivatable (4). Casselli et al. demonstrated that *B. burgdorferi* can colonize the dura mater in mice, are biologically active, and induce host gene inflammatory responses (5). Embers et al. demonstrated post-antibiotic treatment persistence in a non-human primate naturally tick infected model (6) and recovery of the spirochete by xenodiagnosis (2). Similarly, there was recovery of non-cultivatable *B. burgdorferi* by xenodiagnosis in a few human patients who had had an erythema migrans rash and had had prior antibiotic treatment (3). These results, plus observations by us and others that retreatment of patients with recurring or persisting symptoms following initial antibiotic treatment using specific antibiotic regimens (7), lend strong support to the hypothesis that it is persistent infection by *B. burgdorferi* that is the likely cause of persisting symptomatology. In contrast, attribution of post-infectious symptoms to some post-infectious phenomena has only been speculative without any supporting evidence.

## Diagnostic Issues

Currently, in the absence of any currently available means to directly detect the bacteria or its products, the diagnosis is dependent on the clinical history and any associated manifestations, along with the results of serologic studies. A major clinical problem is, that with the exception of patients with Lyme arthritis, most patients with continuing symptoms have no objective signs for Lyme disease, making the diagnosis dependent on the clinical picture that overlaps with that of chronic fatigue syndrome and fibromyalgia. Making it more difficult is the fact that many such patients do not have robust serologic responses to the causative organisms (8). And, despite claims that once one is treated with 4 weeks of antibiotics, one no longer has Lyme disease, or, if Lyme test results revert to negative, it means one no longer has Lyme disease, these claims being unsupportable in the absence of any means to prove the bacteria's absence (9).

It also appears illogical, when patients have persisting or relapsing symptoms identical to those at the initial presentation, to opine that the infection is no longer present and that the remaining symptoms are post-Lyme disease of yet to be defined cause. It would seem more logical to assume that the infection has not been eradicated. It may be that ongoing symptoms are due to post-infectious factors, e.g., autoimmunity without provocation from persistent infection, but that has yet to be demonstrated as an obvious cause of the ongoing clinical picture. It seems much more likely that the cause of symptoms are due to some bacterial product, be it an exotoxin or endotoxin, similar to that that is at the root of most, if not all other bacterial infections, accompanied with host-responses to that virulence product or products, including inflammatory and autoimmune responses (10).

## Serologic Issues

A similar lack of logic is present in analyzing the results of Lyme Western blot reactions, specifically IgM responses. How logical is it to use positive IgM responses to support the diagnosis of early Lyme disease, but deem that those same responses in patients with ongoing or relapsing symptoms are false-positive responses? Is it not more logical to assume that continued IgM reactivity, in the presence of ongoing symptoms, might be an indicator of unresolved infection in the absence of any available test to determine the continuing presence or absence of the causative organisms? In support of that conjecture, the results of several studies in various animal models, indicate that the causative borrelia are able to modulate humoral antibody responses such that the normal conversion of IgM to IgG antibody responses is abrogated (11).

## Treatment Issues

As if confirming the diagnosis isn't sufficiently difficult, the treatment of relapsing or persisting symptoms has presented its own challenges. There are many antibiotics that are active *in vitro* against the Lyme bacteria, but have not been clinically very effective. In early Lyme disease, treatment with doxycycline, amoxicillin, or cefuroxime over a period of a few weeks is generally effective. It is in patients with relapsing or persisting symptoms, including those previously treated, inadequately treated, or untreated, that the question arises as to whether any further antibiotic treatment is effective. The answer appears to be yes, if one looks at the pharmacology of specific antibiotics.

Doxycycline appears to have limited efficacy in patients with persisting or relapsing symptoms, especially in patients with symptoms present for greater than a few months. Doxycycline is highly protein-bound in the circulation, and it is unlikely that sufficient antibiotic can diffuse into tissues and cells to affect the borrelia. In contrast, tetracycline, which is not highly protein bound, appears to be clinically effective (10). Our observational results in several thousands of patients since our initial publication attests to both the greater efficacy of tetracycline vs. doxycycline in terms of both dosing and duration of treatment (12).

Beta-lactam antibiotics, including intravenous ceftriaxone, appear to be of limited clinical efficacy, perhaps because (a) that class of antibiotic has its effects on multiplying organisms, and there is no evidence that the Lyme borrelia are multiplying in persistent or relapsing disease, and (b) they are incapable of intracellular penetration. These antibiotics may offer temporary symptom relief, which might be due to their effects on glutamate accumulation during neurotransmission (13), without resolving the underlying infection.

Of particular interest are the effects of macrolide antibiotics (e.g., erythromycin, clarithromycin, azithromycin) on Lyme disease. They are highly active *in vitro*, and are capable of intracellular penetration, but appear to be of limited clinical value in patients with persistent symptoms. In analyzing the possible reasons, if the borrelia reside intracellularly in an acidic endosome, as is the case for numerous other microbes capable of intracellular persistence, macrolide antibiotics are not very active at an acidic pH. The use of a lysosomotropic agent (e.g., hydroxychloroquine, amantadine) to alkalinize the acidic endosome appears to result in clinical efficacy (14).

There have been two clinical trials using differing antibiotic regimens over a 3 month period of time in patients with persisting symptoms of Lyme disease. In the first trial, patients were given a month of ceftriaxone followed by 2 months of doxycycline vs. placebo treatment, and positive PCR reactivity to *Borrelia burgdorferi* was an exclusionary criterim for this study (15). In the other trial, patients with persisting symptoms were given an initial week of IV ceftriaxone, then randomized to being given the combination of clarithromycin and hydroxychloroquine vs. placebo for 3 months (16). In neither trial was there any reported greater improvement between the antibiotic treatment arms and placebo arms. The results of these studies have been reviewed with several reservations being expressed about study design, the instruments used to measure changes in symptoms, and interpretation of the results (17). In the former trial, neither ceftriaxone nor doxycycline were given for 3 months, and the assumption that both antibiotics are of equal efficacy is not supportable according to differing mechanisms of action. In the case of ceftriaxone, its antibiotic activity is based on its interference with replicating organisms, and given that there is no evidence to indicate that, once *B. burgdorferi* has established itself, there is any multiplication of note, it would not be expected to be effective in patients with persistent symptoms. And in the author's observational experience, even the use of ceftriaxone over periods of time up to 6 months or more was without much if any benefit, with any possible benefit in a few patients due to ceftriaxone's interference with the glutamate receptor system. In the case of doxycycline, whether a longer duration of treatment or increased dosing would have been effective remains unanswered. Observations by numerous clinicians suggest that higher doses of doxycycline, i.e., 300–400 mg/day might be more effective than the commonly used dosing of 200 mg/day.

In the trial utilizing the combination of clarithromycin and hydroxychloroquine, based on our initial published report, the trial was contaminated by the use of ceftriaxone in all patients prior to randomization to the active or placebo groups. Of greater importance is the failure to consider both the duration of prior symptomatology and the duration of treatment itself. As indicated in our published observations (12, 14), patients with persistent or relapsing symptoms for less than a year appeared to be cured, ie no recurring symptoms for greater than a year, by a treatment course of 3–6 months. In patients with persisting symptoms for >2 or more years, however, treatment success required a treatment duration of 6 or more months, and up to 18 months in patients with persisting symptoms for >5 or more years. Another likely flaw in that trial was not controlling for the use of adjunctive vitamin C. Supplemental vitamin C can be a strong acidifying agent, counteracting the effects of hydroxychloroquine (7, 14), and thus possibly accounting for some of the trial's failure to show any benefit of this treatment.

## Future Directions

The key remaining questions are whether there can be found a better, more direct detection test to indicate the presence or absence of active *B. burgdorferi*, and whether additional controlled treatment trials using longer durations of treatment with the tetracycline or clarithromycin/hydroxychloroquine regimen, or regimens utilizing different antibiotics or combination of certain antibiotics that might prove effective. The results of recent *in vitro* and early animal model experiments by Zhang (18) and by Lewis (19) might hold promise of other potentially effective approaches to the management of patients with persistent symptoms of Lyme disease.

Of additional likely importance is the potential role of antibiotic tolerance as a mechanism of persistence and “resistance” of *B.burgdoferi* to treatment in patients with persisting symptoms. Recent results of experiments with other bacterial organisms that can persist demonstrate the likely role of antibiotic-tolerance as the mechanism by which they persist (20). This mechanism apparently relies on a ribonuclease produced by the organisms. If our preliminary results with BB0755, an annotated ribonuclease, that demonstrated cytotoxic activity with tissue-cultured cells of neural origin (21), is due to its ribonuclease activity, then this possibility might offer an explanation to *B.burgdorferi's* antibiotic tolerance.

There are additional questions that a better understanding of the pathophysiology of Lyme disease might lead to better approaches to the diagnosis and treatment of Lyme disease, especially in its persistent form. These include the possible role of antibiotic-tolerant persisters.

## Author Contributions

The author confirms being the sole contributor of this work and has approved it for publication.

## Conflict of Interest

The author declares that the research was conducted in the absence of any commercial or financial relationships that could be construed as a potential conflict of interest.

## Publisher's Note

All claims expressed in this article are solely those of the authors and do not necessarily represent those of their affiliated organizations, or those of the publisher, the editors and the reviewers. Any product that may be evaluated in this article, or claim that may be made by its manufacturer, is not guaranteed or endorsed by the publisher.
